# Value of Galectin-3 assay in children with heart failure secondary to congenital heart diseases: a prospective study

**DOI:** 10.1186/s12887-020-02427-9

**Published:** 2020-11-28

**Authors:** Nagwan Saleh, Ahmed Khattab, Mohamed Rizk, Sherif Salem, Hany Abo-Haded

**Affiliations:** 1 Department of Pediatrics, Faculty of Medicine, Menoufia University, Menoufia, Egypt; 2 Department of Medical Biochemistry, Faculty of Medicine, Menoufia University, Menoufia, Egypt; 3 Pediatric Cardiology Unit, Department of Pediatrics, Faculty of Medicine, Mansoura University, Mansoura, Egypt

**Keywords:** Galectin-3, heart failure, children, congenital heart disease

## Abstract

**Background:**

Galectin-3 is a new biomarker, which plays an important role in tissue inflammation, cardiac remodeling, and fibrosis. It can be readily measured in the circulation to detect early heart failure (HF). This study aimed to assess the value of galectin-3 assay in early diagnosis of children with heart failure secondary to congenital heart disease (CHD) and correlate it with the patients’ outcome.

**Methods:**

This prospective cohort study included 75 children diagnosed to have CHD; {Group A: 45 CHD children with HF symptoms and reduced ejection fraction (REF) and Group B: 30 CHD children with no HF symptoms and normal ejection fraction (NEF)}. They were compared to 40 age- and sex-matched controls (Group C). Children with CHD undergone history taking, Ross HF classification, Echocardiographic assessment and laboratory investigations including serum galactin-3 level.

**Results:**

Galectin-3 serum level increased in CHD children, and it showed significant increase in (Gp A) compared to Gp B or Gp C (*p* = ≤ 0.001). In addition, serum level of Galactin-3 was correlated positively with Ross classification (*r *= 0.68, *p* = 0.018) and negatively correlated to EF% (*r*= -0.61, *p* ≤ 0.001). Galactin-3 showed better diagnostic value than Ross HF classification in early diagnosis of HF in CHD children with a cut point (≥ 10.4), significantly had 96.7% sensitivity, 90% specificity, 91% positive predictive value, 93.2% negative predictive value, with area under the curve (AUC = 0.96) and 93% accuracy. While there was a significant correlation between Ross HF classification and HF outcome in (Gp A) children (*p* = 0.05), we did not find any significant correlation between serum galectin-3 level and HF mortality in same group (*p* = 0.08).

**Conclusions:**

Galectin-3 assay is a promising marker for early diagnosis of HF in children with CHD; but it has no role in detecting HF mortality.

## Background

Congenital heart diseases (CHD) are abnormalities within the heart’s structure that are present since birth. It is the most common birth defect, approximately affecting 1% of all live born infants [1].

Heart failure (HF) in pediatrics represents a serious purpose of morbidity and death in childhood. Clinically, HF usually manifest as shortness of breath or fatigue due to the functional or structural deterioration of ventricular filling or cardiac output [2].

Three putative routes have been proposed for the development of HF in CHD: monogenic entities that cause both CHD and HF; severe CHD lesions in which acquired hemodynamic effects of CHD or surgery result in HF; and, most commonly, a combined effect of complex genetics in overlapping pathways and acquired stressors caused by the primary lesion [3].

The use of cardiac markers is an important way in which suspected children can be assessed for the existence of and/or grade of HF [4]. Galectin-3, a soluble beta-galactoside-binding lectin, is subtle in cardiomyocytes. In response to myocardial injury, it freed by cardiac macrophages, provokes fibroblast increase and deposition of collagen in the cardiac muscle by inducing growth factor beta (TGF-b) and exciting matrix production [5, 6].

A developing proof that galectin-3 is mainly associated with the pathology of cardiac insult and the development of HF, making it a likely early diagnostic, predictive and therapeutic marker [7, 8].

This study aimed to assess the value of galectin-3 assay in early diagnosis of children with heart failure secondary to CHD and correlate it with the patient’s outcome (mortality).

## Methods

### Study population

A prospective cohort study was carried at pediatric department, in Menoufia University, Egypt during the period from October 2018 to September 2019. According to the number of populations in the locality, the prevalence of children with CHD and the number of patients following-up in the pediatric cardiology clinic, a sample of 75 children known to have CHD was considered. These children were divided into two groups; Group (A) which included 45 children who were subsequently admitted with HF symptoms and reduced ejection fraction (REF) by Echo [24 (53.3%) males and 21 (46.7%) females; age range: 0.6–9.3 years, mean age: 3.7 ± 2.89 years]. Then, Group (B) which included 30 children who were following-up in the cardiology outpatient clinic with no HF symptoms and normal ejection fraction (NEF) by Echo [13 (43.3%) females and 17 (56.7%) males; age range: 0.8 − 9.6 years, mean age: 3.9 ± 2.01 years]. Both groups were compared to 40 age- and sex-matched controls (Group C) [20 (50%) females and 20 (50%) males; age range: 0.5– 10.3 years, mean age: 5.1 ± 3.95 years].

Exclusion criteria were age ≥ 18 years, affected renal function (Glomerular filtration rate (GFR) ≤ 70 ml/min/1.73 m2), primary hepatic and lung diseases, (e.g., hepatic cirrhosis and lung fibrosis, multiple congenital anomalies, or heart failure secondary to cardiomyopathy).

All children exposed to thorough history taking documenting heart failure symptoms, detailed clinical examination and signs of heart failure were recorded. Staging of the heart failure was performed per the “symptom-based Modified Ross Heart Failure Classification for Children” [9].

Investigations included Vivid-7 echocardiography system (GEMedical Systems, Waukesha, Wisconsin, USA) per the suggestions of the American Society of Echocardiography. Apical chamber views and M-mode were used for left ventricular end diastolic volume (LVEDD) and left ventricular end systolic volume (LVESD); left ventricular ejection fraction (EF %) and fractional Shortening (FS %) were determined with modified Simpson’s method using biplane apical (2- and 4-chamber) views. All the echocardiography examinations were done by expert pediatric cardiologists blinded to the results of the serum biomarker assay. To evaluate intra-observer changeability, echocardiography data from video recordings of 15 randomly chosen children were examined twice by the same cardiologist within a period of time between the two observations around 10 days interval; the intra-observer changeability in the assessment of EF % and FS % was found to be < 5%.

Laboratory tests included, complete blood count (CBC) assessment, kidney function tests (serum creatinine, GFR). For the assay of serum galectin-3, blood samples were collected in plastic syringes, and rapidly conveyed to chilled tubes. The serum was separated by centrifugation at 4 °C for 20 min and stored at -80 °C. Serum galactin-3 was measured using ELISA kit that is manufactured for research use only (SunRed Bio-technology Co., Shanghai, China). Briefly, serum was incubated with galactin-3-HRP conjugate for 1 h followed by washing. A blue colored product was obtained after incubation with a substrate for HRP enzyme, which turned into yellow color after adding the stop solution. The intensity of color was measured spectrophotometrically at 450 nm. Sensitivity was 0.24 ng/ml, the intra-assay and inter-assay reproducibility coefficient of variation was 7.8% & 5.4% respectively, and linearity range 94–109%. The children’s clinical data were dumped from the staff at the laboratory facility.

### Statistical analysis

Data were collected, organized and statistically analyzed using statistical Package of Social Science (SPSS) version 20. Quantitative data were expressed as mean ± standard deviation (SD). Qualitative data were expressed as frequency and percentage. The following tests were done as appropriate: Chi-squared test (χ2), student t-test (T), Kruskal wails test (K), one-way ANOVA test (F), Mann-Whitney test (U). Spearman correlation’s correlation coefficient (r) test was used for correlating data. *p value* ≤ 0.05 considered statistically significant.

## Results

The demographic, clinical and laboratory characteristics of the studied population are summarized in Table 1. There was no significant difference found between the studied groups regarding age, sex, weight, and systolic blood pressure. On the other hand, there was statistical significant difference between the three groups as regard height, and BMI, heart rate, respiratory rate (*p value* was 0.031, 0.032, ≤ 0.001, and ≤ 0.001, respectively).

**Table 1:** Demographic, clinical, echocardiographic findings, and laboratory investigations of the studied groups

Data	Group A (No.=45)	Group B (No =30)	Group C (No=40)	Test of significance	***P*** value
**Clinico-demographic data**					
**Sex**					
Male no. (%)	24 (53.3)	17 (56.7)	20 (50)	**χ**^**2**^(0.03)	0.95
Female no. (%)	21 (46.7)	13 (43.3)	20 (50)		
**Age(years)**					
Mean ± SD	3.7 ± 2.89	3.9 ± 2.01	5.1 ± 3.95	K (0.88)	0.76
Range	0.6-9.3	0.8-9.6	0.5– 10.3		
**Weight (Kg)**					
Mean ± SD	8.35±3.9	8.5±3.8	9.8±4.7	K (0.95)	0.32
**Height (cm)**					
Mean ± SD	65.23 ± 15.9	70.8 ±16.2	72.2±22.4	F (0.61)	0.031*
**BMI (Kg/m2)**					
Mean ± SD	13.1± 2.9	14.4±2.6	16.7±2.3	F (0.60)	0.032*
**HR**					
Mean ± SD	127.5±17.1	81.4±9.3	70.3±6.7	F (159.2)	≤0.001*
**Post Hoc :** P1=≤0.001, P2=≤0.001, P3=0.03					
**RR**					
Mean ± SD	46.5±13.4	37.5±6.5	35±7.1	F (10.1)	≤0.001*
**Post Hoc :** P4≤0.001, P5=≤0.001, P6=0.36					
**Systolic blood pressure**					
Mean ± SD	93 ± 12.1	93.3 ±9.8	87±5.4	F (2.9)	0.06
**Echo Findings**					
**Ejection fraction %**					
Mean ± SD	36.9±3.9	61.1±5.2	NA	T (36.8)	≤0.001*
Range	32.8 – 40.8	55.7 – 66.9			
**Fractional Shortening %**					
Mean ± SD	23.3±4.1	42.4±3.2	NA	T (20.1)	≤0.001*
Range	18.8 – 28.5	37.6 – 45.1			
**LVEDD**					
Mean ± SD	32.6± 11.1	29.1±3.1	NA	T (1.68)	0.0971
**LVESD**					
Mean ± SD	23.7± 6.7	13.7±2.3	NA	T (7.85)	≤0.001*
**laboratory investigations**					
**Hb (gm/dl)**					
Mean ± SD	10.1±1.4	9.9±1.4	10.1±1.3	F ( 0.39)	0.67
**Leucocyte count /cm3**					
Mean ± SD	13.3± 3.9	13.7±3.7	7.4±3.7	K (24.4)	≤0.001*
**Platelet/cm3**					
Mean ± SD	240.6±62.2	236.7±61.2	236.4±59.2	F (0.04)	0.96
**Serum creatinine**					
Mean ± SD	0.59± 0.25	0.54±0.22	0.48±0.12	K (0.42)	0.0516
**Galectin-3 (ng/dl)**					
Mean ± SD	20.3±5.3	6.3±4.4	3.08±1.5	K (50.7)	≤0.001*
**Post Hoc:** P7=≤0.001, P8=≤0.001, P9=0.01					

*Statistical significance

P1=comparison of HR between Group A and Group B, P2=comparison of HR between Group A and Group C

P3=comparison of HR between Group B and Group C

P4=comparison of RR between Group A and Group B, P5=comparison of RR between Group A and Group C

P6=comparison of RR between Group B and Group C

P7=comparison of serum galectin-3 between Group A and Group B, P8=comparison of serum galectin-3 between Group A and Group C, P9=comparison of serum galectin-3 between Group B and Control group

**Fig. 1:**
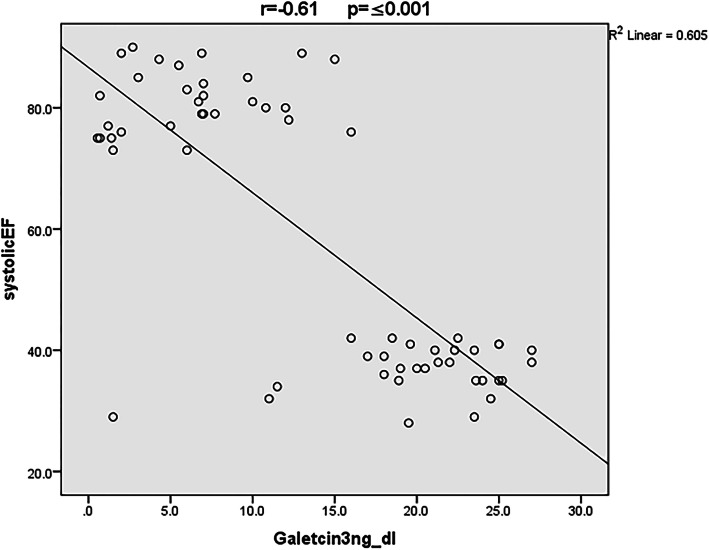
Correlation between serum Galectin-3 level and EF%

Echo measurements in children with CHD (Group A & B) showed significant difference between both groups regarding EF%, FS%, and LVESD (*p value* was ≤ 0.001). A significant negative correlation was found between serum galectin-3 levels with EF% and FS% (r= -0.61, *p value* ≤ 0.001, and r= -0.66, *p value* ≤ 0.001, respectively). While there was a positive correlation between Galectin-3 serum level with LVESD (r = 0.69, *p value* ≤ 0.003), but no correlation with LVEDD was reported (r= -0.04, *p value* = 0.821). The negative correlation between serum galectin-3 and EF% is shown in Fig. 1.

There were statistical significant difference between the three groups regarding total leucocyte count (*p value* was ≤ 0.001). In addition, Galectin-3 serum level increased in children with CHD, and it showed significant increase in group (A) when compared to the both groups (B & C), *p value* ≤ 0.001.

Table 2 showed the detailed types and statistical significance between different congenital heart diseases in the study population (Group A versus Group B, acyanotic versus cyanotic subtypes).

**Table 2:** The detailed types and statistical significance between different congenital heart diseases in the study population (Group A versus Group B, acyanotic versus cyanotic subtypes)

Echo Findings	Group A (=45 children) No. (%)	Group B (=30 children) No. (%)	Test of significance	***p value***
**Congenital acyanotic heart diseases**				
**ASD**	6 (13.3 %)	4 (13.3 %)	**χ**^**2**^(0.000)	1.0000
**VSD**	14 (31.1 %)	10 (33.4 %)	**χ**^**2**^(0.04085)	0.8398
**PDA**	7 (15.6 %)	5 (16.7 %)	**χ**^**2**^(0.01653)	0.8977
**ASD+VSD**	4 (8.9 %)	4 (13.3 %)	**χ**^**2**^(0.05247)	0.8188
**PS**	8 (17.8 %)	5 (16.7 %)	**χ**^**2**^(0.01551)	0.9009
**Congenital MR**	1 (2.2 %)	0 (0 %)	**χ**^**2**^(0.6757)	0.4111
**Galectin-3 (ng/dl) in acyanotic heart diseases**				
Mean ± SD	**20.1±4.9**	**5.8±4.9**	T (12.382)	< 0.0001*
**Congenital cyanotic heart diseases**				
**TGA**	2 (4.4 %)	1 (3.3 %)	**χ**^**2**^(0.05787)	0.8099
**TOF**	3 (6.7 %)	1 (3.3 %)	**χ**^**2**^(0.01100)	0.9165
**Galectin-3 (ng/dl) in cyanotic heart diseases**				
Mean ± SD	**19.9±5.1**	**6.2±4.3**	T (12.114)	< 0.0001*
***p value***	P1= 0.8500	P2= 0.7380	**-**	-

*Statistical significance

χ2= Chi-squared test with Yate’s continuity correction, T= student t-test

P1=comparison of serum galectin-3 between acyanotic and cyanotic heart disease (in Group A)

P2=comparison of serum galectin-3 between acyanotic and cyanotic heart disease (in Group B)

*ASD* atrial septal defect, *VSD* ventricular septal defect, *PDA* patent ductus arteriosus, *PS* pulmonary stenosis, *MR* mitral regurgitation, *TGA* transposition of great arteries, *TOF* tetralogy of fallot

**Fig. 2:**
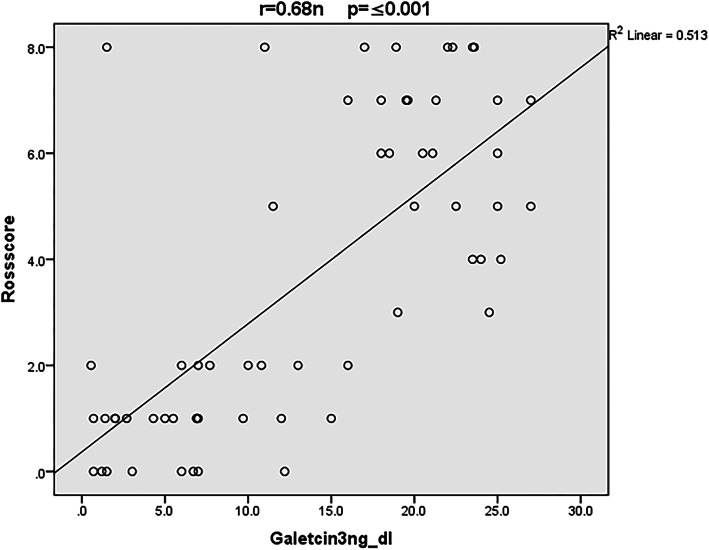
Correlation between serum Galectin-3 level and Ross HF classification score

Figure 2 showed the positive correlation between Galectin-3 serum level with Ross HF classification score (*r* = 0.68, *p value* ≤ 0.001).

**Fig. 3:**
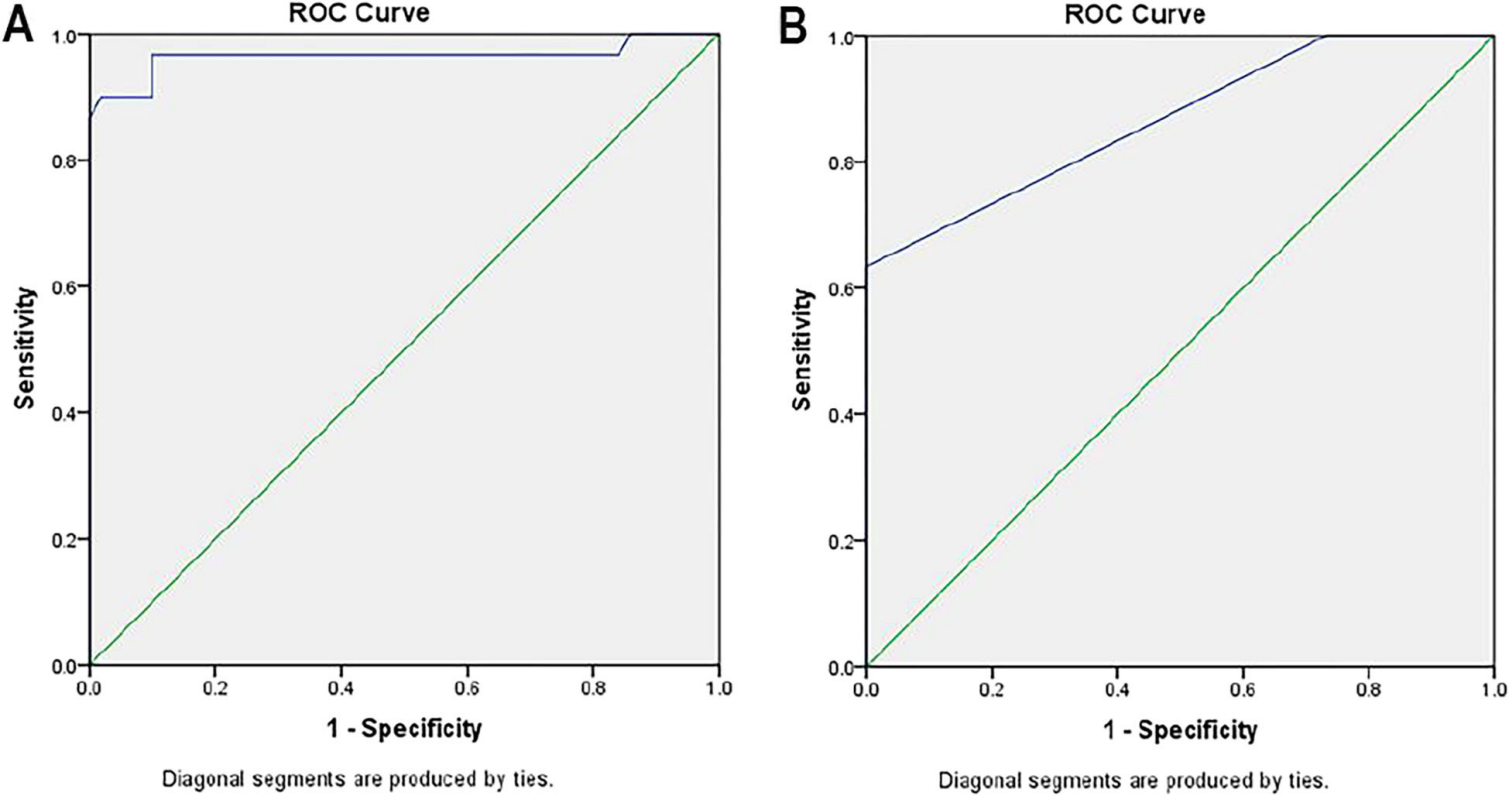
Roc curve of: (**a**) Serum Galectin-3 level, (**b**) Ross score

The validity of galectin-3 assay and Ross HF classification score for the early diagnosis of HF in children with CHD are compared (Table 3; Fig. 3). Serum Galectin-3 at cut point (≥ 10.4) significantly had 96.7% sensitivity (*p value* = 0.028), 90% specificity, 91% positive predictive value, 93.2% negative predictive value, with area under the curve (AUC = 0.96) and 93% accuracy; while Ross score at cutoff point (≥ 1.5) significantly (*p value* = 0.04) has 83.3% sensitivity, 74.3% specificity, 72.8% positive predictive value, 82.1% negative predictive value, with area under the curve (AUC = 0.86) and 78% accuracy in diagnosis of heart failure of patients with congenital heart disease.

**Table 3:** Comparing the validity of Galectin-3 assay and Ross HF classification for the early diagnosis of heart failure in children with congenital heart disease

Test	AUC	***P*** value	Cutoff point	Sensitivity	Specificity	NPV	PPV	Accuracy
**Galectin-3 (ng/dl)**	0.96	0.028	≥10.4	96.7%	90%	93.2%	91%	93%
**Ross Score**	0.86	0.04	≥1.5	83.3%	74.3%	82.1%	72.8%	78%

*AUC* area under the curve, *NPV* negative predictive value, *PPV* positive predictive value

A follow-up of the outcome of the CHD children at the end of the study was done. Group (A) children presenting with HF and REF {33 children were controlled on medication, symptoms of HF improved and normal ejection fraction (NEF) were restored; on the other hand, 12 children showed more deterioration of the presenting HF symptoms, no response to medical treatment, and finally passed away}. On the other hand, Group (B) children presenting with NEF showed no significant morbidity or mortality during the study period.

A comparison was held between serum galectin-3 and Ross HF classification score at presentation with the outcome (mortality) of CHD children (Group A). While, no statistical significant were found between Ross HF classification score at presentation and the outcome of CHD children (*p value* = 0.2379), there was a statistical significance regarding early presenting galectin-3 serum level and mortality of CHD children (*p value* = 0.0011), (Table 4).

**Table 4:** Comparison between serum galectin-3 level & Ross HF classification with the outcome of CHD children presenting with HF symptoms (Group A = 45 children)

Variables	Improved Children (No. = 33)	Died Children (No. = 12)	Test of significance	***P*** value
**Galectin-3 (ng/dl)**				
Mean ± SD	10.1±5.4	17.4±8.1	(U) 3.49	0.0011*
Range	0.7-12.7	1.6-27		
**Ross score**				
Mean ± SD	3.1±2.8	4.2±2.5	(U) 1.197	0.2379
Range	0-8	0-8		

*U* Mann-Whitney test

*Statistical significance

## Discussion

Congestive heart failure is a serious condition where the heart can’t pump efficient blood to supply metabolic demands. Over time, the inefficient cardiac output triggers a cascade of compensatory mechanisms aiming directly or indirectly at maintaining normal perfusion to vital organs and tissues [10]. An approximate of 5–25% of children with structural heart disease develop HF [11].

Galectin-3 has been increasingly identifiable as a major modulator of different biological reactions, by co-interacting with several molecules both inside and outside the cell .Tissue fibrosis is a major contributing factor in the developing of heart failure. Fibrosis deteriorates cardiac function and significantly promotes to both systolic and diastolic dysfunction. Studies have shown that galectin-3 as a biomarker plays a significant part in tissue fibrosis and ventricular remodeling. It is highly expressed inside cardiac fibroblasts [12].

This study agrees with El-Amrousy, et al. study who found that there was no significant difference between heart failure group and control group in age, sex, or body weight [13], But a statistical significance between the studied groups regarding height, and BMI was reported, which agrees with Rubia and Kher study who demonstrated that there is an evidence of growth retardation and hence BMI in children with CHD [14]. Schwartz, et al. found that decreased energy intake, malabsorption and increased basal energy requirements might all lead to compromised growth and underweight in children with CHD [15].

This study found that there was statistical significant difference between the three groups as regard heart rate and respiratory rate. This agreed to the studies of El-Amrousy, et al., and with Masatsugu & Hiroshi in which their studies found that heart rate and respiratory rate are mainly regulated by autonomic nervous activities; specifically increased with attenuated vagal nerve activity or enhanced sympathetic nerve action in children with HF [13, 16].

Mohammed et al. who found a significant positive correlation between galectin-3 levels with LVESD and LVEDD, indicating an early detection of heart failure before any functional changes, and a negative correlation was detected between galectin-3 levels with FS% and EF%. His findings are concordant with our study, except for the LVEDD; as we didn’t find any correlation with LVEDD. The previous study considered that the correlation between the clinical and cardiac changes with galectin-3 level give an indirect proof supporting a probable function for galectin-3 in the pathogenesis of cardiac remodeling in HF, and demonstrated that after an early injury to the cardiac muscles compensatory mechanism of cardiac remodeling occurs, leading to left ventricular dysfunction and HF [17].

Iqbal, et al. study showed that there was significant evidence of cardiac remodeling and worsening of EF% -detected by echocardiography- between cardiac and healthy children. They considered that the cardiac structural changes - in the form of inflammation & fibrosis - and the clinical worsening correlations with galectin-3 level were considered as an indirect supporting evidence of a significant action of galectin-3 in the substantial pathogenesis of heart failure [18]. Also, similar to our study. Kotby, et al. study showed a strong correlation between the level of galectin-3 and EF% (*p value* ≤ 0.001) [19].

This study also found significant increase in total leucocyte count, which is considered as an innate marker of acute or chronic systemic inflammation. Also, total leucocyte count act as a risk marker for cardiovascular disease. Engström, et al. studies showed that moderate increase of leukocyte concentrations is associated with incidence of hospitalizations due to HF [20].

There was mild non-significant increase in creatinine level in CHD children. This was in line with Shlipak, et al. study that have declared the value of measuring kidney function in heart failure. The increased creatinine levels while hospitalized is a marker of low cardiac output that contributes to decreased renal vascular flow and decreased ability to bear the inpatient heart failure management. Thus, the change in creatinine level is mostly indicative of the severity of cardiac muscle dysfunction instead of acute kidney damage [21].

There was a significant increase of serum galectin-3 level between the studied groups mainly in CHD children with HF (Group A). This was in a line with Kotby, et al. and Meeusen, et al. studies who found that children with HF had a significant elevation of galectin-3 serum level in comparison to controls with mean galectin-3 of (5.75 ± 1.427 ng/ml) at control group (*P* value ≤ 0.001) compared to mean of (18.40 ± 11.5 ng/ml) at HF patients [19, 22]. Numano, et al. explanation is that the myocyte insult induces multiple inflammatory mediators like osteopontin and these mediators stimulate the activated macrophages to secrete galectin-3 [23].

A positive correlation was noticed between serum galectin-3 level and the Ross HF classification score. This can be interpreted by the fact that apoptosis is concerned in the transition to decompensated HF, and since galectin-3 is entangled in apoptosis; thus, the increase in galectin-3 levels is may be prompted to heart failure decompensation [24].

Galactin-3 showed a better diagnostic value than Ross HF classification score in early diagnosis of HF in CHD children. This may be due to the concentrations of galectin-3 have been linked to markers of extracellular matrix turnover, including a correlation between macrophage activation and collagen turnover. In addition, the clinical manifestations of heart failure may be preceded many years by active fibrosis, so the presentation of high galectin-3 level may occur before manifest HF and thus may be more effective for the prevention and prediction of disease sequelae [17, 25].

The study outcome (mortality) percentage of CHD with or without heart failure was in agreement with Moussa, et al. and Zomer, et al. studies who found that HF is a significant event in CHD and is associated with a high mortality rate (20%). They found mortality five-fold more in CHD children with HF in comparison with patients without. Moreover, hospitalization due to HF was related to a significant increase in the risk of cardiovascular events [26, 27]. However, to date, this study is the first one to report the correlation between the presenting serum Galactin-3 and prediction of mortality in the HF group. Limitation of the study: Serial measurements of serum galectin-3 levels were not performed.

## Conclusions

Galectin-3 has been emerged as an evolving biomarker for early diagnosis of HF in children with CHD; and has a valuable predictive role for the patient’s outcome.

## Data Availability

Readers can get the datasets and materials of the current study by contacting the corresponding author of Hany Abo-Haded (hany_haded@yahoo.com) for a reasonable request.

## Abbreviations

CHD: Congenital heart diseases
EF: Ejection fraction
FS: Fractional Shortening
HF: Heart fraction
NEF: Normal ejection fraction
REF: Reduced ejection fraction
ROC: Receiver operating characteristic
